# The Obesity Paradox in Cancer, Tumor Immunology, and Immunotherapy: Potential Therapeutic Implications in Triple Negative Breast Cancer

**DOI:** 10.3389/fimmu.2019.01940

**Published:** 2019-08-14

**Authors:** Adviti Naik, Arta Monir Monjazeb, Julie Decock

**Affiliations:** ^1^Qatar Foundation (QF), Cancer Research Center, Qatar Biomedical Research Institute (QBRI), Hamad Bin Khalifa University (HBKU), Doha, Qatar; ^2^Department of Radiation Oncology, UC Davis Comprehensive Cancer Center, University of California, Sacramento, Sacramento, CA, United States

**Keywords:** obesity, breast cancer, triple negative breast cancer, immunotherapy, meta-inflammation

## Abstract

Cancer immunotherapy has been heralded as a breakthrough cancer treatment demonstrating tremendous success in improving tumor responses and survival of patients with hematological cancers and solid tumors. This novel promising treatment approach has in particular triggered optimism for triple negative breast cancer (TNBC) treatment, a subtype of breast cancer with distinct clinical features and poor clinical outcome. In early 2019, the FDA granted the first approval of immune checkpoint therapy, targeting PD-L1 (Atezolizumab) in combination with chemotherapy for the treatment of patients with locally advanced or metastatic PD-L1 positive TNBC. The efficacy of immuno-based interventions varies across cancer types and patient cohorts, which is attributed to a variety of lifestyle, clinical, and pathological factors. For instance, obesity has emerged as a risk factor for a dampened anti-tumor immune response and increased risk of immunotherapy-induced immune-related adverse events (irAEs) but has also been linked to improved outcomes with checkpoint blockade. Given the breadth of the rising global obesity epidemic, it is imperative to gain insight into the immunomodulatory effects of obesity in the peripheral circulation and within the tumor microenvironment. In this review, we resolve the impact of obesity on breast tumorigenesis and progression on the one hand, and on the immune contexture on the other hand. Finally, we speculate on the potential implications of obesity on immunotherapy response in breast cancer. This review clearly highlights the need for *in vivo* obese cancer models and representative clinical cohorts for evaluation of immunotherapy efficacy.

## Introduction

In 2014, the World Health Organization (WHO) reported that cancer is the second deadliest non-communicable chronic disease and that the number of new cases and cancer-related deaths would increase over the next decade. Breast cancer remains the most common cancer among women, accounting for 30% of all cancer cases (1). Moreover, according to the International Agency for Research on Cancer (IARC), breast cancer has become the leading cause of female cancer-related deaths. Triple negative breast cancer (TNBC), representing 15–20% of breast tumors, is associated with excess mortality accounting for almost one third of breast-cancer related deaths (2). TNBC patients face a poor clinical outcome with an early relapse peak at 3–5 years after diagnosis (3). Due to the lack of expression of the estrogen receptor (ER), progesterone receptor (PR), and the human epidermal growth factor receptor (Her2), until recently, cytotoxic chemotherapy was the only approved systemic treatment option for TNBC. Although these patients achieve more pathologic complete responses compared to other breast cancer patients, their recurrence and metastasis rates are far greater (4).

Given the success of immunotherapy in acute lymphoblastic leukemia and melanoma, numerous clinical trials in breast cancer have sprouted (5, 6). Breast tumors were considered immunologically cold; however, it has become clear that certain subtypes could benefit from immunotherapy. Immune cell infiltration has been observed in hormone receptor negative breast tumors, and enumeration of tumor infiltrating lymphocytes (TILs) positively correlates with clinical outcome and treatment response in TNBC (7). In addition, expression of the immune checkpoint ligand Programmed Death Ligand 1 (PD-L1) in TNBC tumors suggests that inhibiting its interaction with the Programmed Death-1 (PD-1) receptor could restore T cell activation and support anti-tumor immunity (8). In metastatic setting, PD-1/PD-L1 blockade results in durable overall response rates of 19%, which increases to 39–54% by addition of chemotherapy (9, 10). In early stage disease, clinical trials report pathological complete response rates of 20–60% using combinatorial and/or concurrent treatments of PD-L1 inhibition with chemotherapy (11). Of note, the FDA recently granted accelerated approval to PD-L1 blockade (Atezolizumab) in combination with nab-paclitaxel as first-line treatment for advanced TNBC. Furthermore, inhibition of the cytotoxic T lymphocyte-associated antigen 4 (CTLA-4) checkpoint is currently under study in two clinical trials (12). Besides immune checkpoint blockade, monoclonal antibodies, cancer vaccines, oncolytic virotherapy, and adoptive T-cell therapy have recently entered clinical trials in TNBC (12).

Unfortunately, clinical benefit from immunotherapy in solid cancers remains limited due to loss of target expression, metabolic reprogramming, impaired T cell trafficking and activation, poor T cell persistence, and induction of an immunosuppressive microenvironment (13). Several risk factors have been associated with low response rates and immune-related adverse events (irAEs) including a low mutational load and family history of autoimmune disorders (14). We postulate that obesity-associated inflammation can significantly dysregulate the immune response and have profound effects on the toxicity and efficacy of immunotherapy. While obese patients tend to respond better to checkpoint blockade, in part by reversing immune suppression, an increase in body mass index (BMI) has been linked to irAEs thereby limiting the therapeutic window (15). According to the WHO global estimates of 2016, about 40% of adults are overweight and 13% obese, and ~18% of children and young adolescents (below 18 years) are overweight or obese. These staggering statistics provide evidence of an obesity epidemic and warrant for its role in numerous diseases including cancer. In this review, we will discuss the implications of obesity on breast cancer, anti-tumor immunity, and immunotherapy efficiency with an emphasis on TNBC.

## Obesity and Breast Cancer Risk

Obesity (BMI ≥ 30 kg/m^2^) is closely linked to the occurrence of metabolic disorders. Excess fat triggers extensive remodeling of the adipose tissue microenvironment in terms of its size, vascularity, and cellular and matrix composition; which is accompanied by deregulated secretion of adipose cytokines or so-called adipokines. Pro-inflammatory adipokines such as leptin, Tumor Necrosis Factor-alpha (TNF-α), interleukin (IL)-6, IL-1, IL-8, and resistin are increased in obesity and are associated with insulin resistance, type 2 diabetes and cardiovascular disorders. On the contrary, anti-inflammatory adipokines such as adiponectin, IL-10, and secreted frizzled-related protein 5 (SFRP5) are downregulated in obesity (16). In addition, increased expression of insulin-like growth factor-1 (IGF-1) and IGF-binding proteins has been observed in obesity as a result of insulin resistance and increased circulating estrogen. It is important to note that such hormone and adipokine imbalances can collectively promote mitogenic and mutagenic pathways, leading to an increased risk of cancer (17).

According to the Million Women Study, obesity contributes to 5% of all cancers in postmenopausal women (18). More specifically, postmenopausal obesity is directly associated with ER+ breast cancer risk whereas ER- or TNBC is minimally or inversely associated with obesity. The most likely explanation for this risk association relates to adipose estrogen pools that stimulate the growth and survival of ER+ tumors in the absence of ovary-derived estrogen. Studies on premenopausal breast cancer risk show a degree of inconsistency with null or lower risk of ER+ breast cancer and higher risk of TNBC with obesity (19). Furthermore, obesity has been associated with increased risk of local recurrence and metastasis, and worse survival in breast cancer (20, 21), particularly TNBC (22). In addition to breast cancer risk, obesity has also been linked to treatment response. For instance, adverse events such as lymphedema, cancer-related fatigue, and poorer quality of life have been linked to obesity (23). It is likely that treatment-related cardiotoxicity involving metabolic and inflammatory abnormalities are exacerbated in obese cancer patients, although this needs to be investigated in further detail. Since immunotherapy is an emerging anti-cancer treatment strategy, it is pivotal to gain insight into possible deleterious effects of obesity on immunotherapy tolerance and response.

## Deregulated Immune Response in Obese Breast Cancer Patients

**Figure 1:**
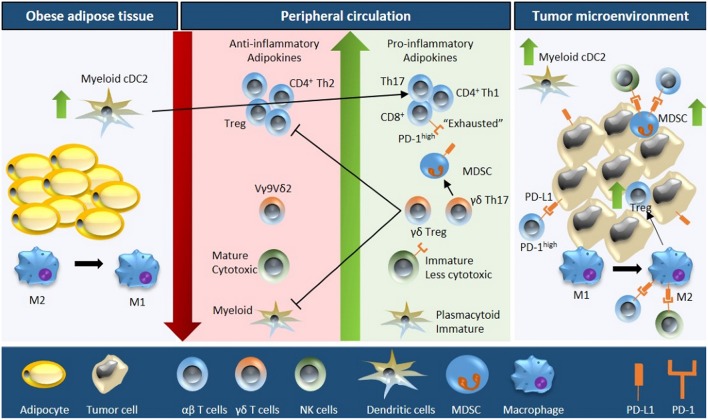
Schematic overview of obesity-associated immune modulations in cancer. Fat accumulation in adipocytes triggers a pro-inflammatory microenvironment within the adipose tissue characterized by M2 to M1 macrophage polarization and accumulation of myeloid conventional DC2 (cDC2) cells. Obese adipose tissue increases the secretion of pro-inflammatory adipokines and free fatty acids concomitant with a downregulation of anti-inflammatory adipokines into the peripheral circulation. As a consequence, the number and activity of cytotoxic T cells decreases (due to reduced proliferation, increased apoptosis, and impaired function of the progenitor thymocytes), NK cell maturation is defective, the plasmacytoid to myeloid DC ratio increases, the number of MDSCs is upregulated, and Vγ9Vδ2 cells are polarized into γδ Treg and γδ T17 cells (further inhibiting the function of cytotoxic T cells and myeloid DCs). Moreover, the obese microenvironment increases the expression of PD-1 on T cells and NK cells, and of PD-L1 on MDSCs. These systemic alterations ultimately result in increased immune evasion, especially due to the interaction of PD-1/PD-L1, increased tumor MDSC and Treg infiltration, and M1 to M2 macrophage polarization; resulting in an immunosuppressive tumor microenvironment.

**Figure 2:**
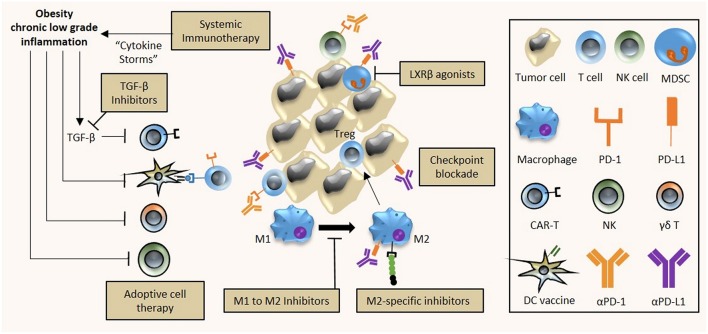
Implications of obesity on cancer immunotherapy. The chronic low inflammatory state associated with obesity has diverse effects on tumor immunity and immunotherapy efficacy. First, the use of systemic immunotherapy in obese cancer patients with meta-inflammation might be contraindicative due to treatment-induced cytokine storms and excessive immune-related adverse events. Second, the efficacy of DC vaccines and adoptive cell therapy (T and NK cells) is hampered by obesity as a result of reduced activity of CAR-T cells; and reduced activity and altered polarization of γδ T cells, NK cells and DC cells into their respective immunosuppressive counterparts. Third, obesity-associated immune alterations provide potential targets that can be exploited for treatment. Obesity-associated increased expression of PD-1/PD-L1 on immune cells can be targeted by checkpoint inhibitors (αPD-1, αPD-L1), M2 polarization of obesity-associated M1 macrophages can be prevented by specific inhibitors while M2 macrophage activity can be inhibited, and apoptosis of the obesity-mediated accumulation of MDSCs can be induced by LXRβ agonists. A combinatorial approach to immunotherapy may be necessary in obese cancer patients, comprising checkpoint blockade, adoptive cell therapy, DC cancer vaccines, and TGF-β inhibition to improve the overall anti-tumor immune response.

It is well-known that obesity results in severe modulation of the immune landscape, leading to a chronically activated immune response. This “meta-inflammatory” state can impair anti-tumor immunity and affect immunotherapy efficacy (24, 25). Hence, it is critical to highlight the differences in the tumor immune microenvironment of obese and lean patients. We will describe the impact of obesity on various immune components in and out of the context of cancer (Figure 1, Table 1); and discuss how this may affect immunotherapy response (Figure 2, Table 2). Wherever possible, we will highlight studies in TNBC.

**Table 1:** Immune modulations in obesity and in cancer.

**Cell type**	**Subtype**	**Main functions**	**Modulations in obesity**	**Modulations in cancer**	**References**
T cells	Thymocytes	T cell progenitor pool	Reduced proliferation and function Increased apoptosis	N/A	(26, 28–31)
	CD8+ Th1 CD4+ Th17 CD4+	Pro-inflammatory	↑ Exhausted activity Increased PD-1 expression	↓ Reduced activation and IFN-γ production Increased checkpoint activation (PD-1/PD-L1)	
	Th2 CD4+ Tregs	Immunosuppressive	↓ Reduced IL-10, increased IFN-γ production	↑	
Γδ T cells	Vγ9Vδ2	Anti-tumorigenic	↓ Reduced IFN-γ production	↓	(38, 39, 41–43, 45)
	Vγ4 and Vγ6 γδ cells	Pro-inflammatory	↑	N/A	
	FoxP3+ γδ Tregs γδ T17 cells	Pro-tumorigenic Immunosuppressive	↑	↑ In advanced disease	
NK Cells	CD56^dim^CD16^dim/−^	Non-cytotoxic Immunosuppressive	N/A	↑ In poor prognosis TNBC Increased PD-1 expression Increased expression of inhibitory receptors	(46–48, 50–54)
	CD56^dim^CD16^bright^	Cytotoxic	↓	↓ In poor prognosis TNBC Decreased expression of activating receptors	
	CD56^bright^	Pro-inflammatory	↑	N/A	
	NKT cells	Immunosurveillance	↓ Reduced IFN-γ production, decreased markers of cytotoxicity	↓	
DCs	Myeloid	Pro-inflammatory	↑ Compromised functionality, maturation	↓	(58, 59, 62, 63)
	Plasmacytoid	Immunosuppressive	N/A	↑ In TNBC Dampened IFN-γ production	
Macrophages	M1	Pro-inflammatory	↑	↓	(64–67, 71, 73, 74)
	M2	Pro-tumorigenic Immunosuppressive	↓	↑ Express PD-L1/PD-L2 and CD80/86 Correlated with poor clinical outcome in TNBC	
MDSC	M-MDSC, PMN-MDSC	Immunosuppressive	↑ Increased PD-L1 expression	↑	(78, 79, 81–83, 85, 88)

*DC, dendritic cells; MDSC, myeloid-derived suppressor cells; M-MDSC, monocytic MDSC; PMN-MDSC, polymorphonuclear MDSC; N/A, not available; NK, natural killer cells; PD-1, programmed death-1; PD-L1, programmed death ligand 1; Th, T helper cell; TNBC, triple negative breast cancer; Treg, T regulatory cells*.

**Table 2:** Immunotherapy implications in obese cancer patients.

**Cell type**	**Reported findings on treatment implications in obese cancer patients**	**References**
T cells	**PD-1/PD-L1 checkpoint inhibition** Increased TILs, CD8+/CD4+ ratio, and improved response and survival. No irAEs observed. **CAR T-cell therapy** Impaired activity and proliferation of CAR T-cells by TGF-β, indicating potential benefit of combination treatment with TGF-β antagonists, in addition to PD-1/PD-L1 checkpoint inhibition.	(29, 32, 97, 99)
γδ T cells	**γδ adoptive immunotherapy or Vγ9Vδ2 agonists** Low efficacy in breast cancer due to γδ T cell exhaustion and plasticity. Potential detrimental effects due to induction of immunosuppressive γδ T cells by obese microenvironmental cues.	(38, 40)
NK cells	**PD-1/PD-L1 checkpoint blockade** Reduction in tumor burden. **Adoptive iNKT cell therapy and IL-6Ra+ NK cell ablation** Reduction in obesity, hyperlipidemia and leptin production, and upregulation of anti-inflammatory cytokines. Potential improved treatment response when combining checkpoint blockade with adoptive NK cell therapy.	(55–57)
DCs	**DC-based immunotherapy** Reduced efficacy in obese renal carcinoma model, associated with increased regulatory DCs and decreased CD8+ T cells. Potential improved response on combining DC-based immunotherapy with obesity interventions.	(61, 63)
Macrophages	**TAM-specific depletion (M2pep, anti-MARCO) or reprogramming (epigenetic modulators)** Improved treatment response (pre-clinical) in combination with checkpoint blockade (anti-PD-1/PD-L1, anti-CTLA4).	(68–70, 72)
MDSCs	**LXRβ agonists (GW3965, RGX-104a)** Induction of MDSC apoptosis and improved anti-tumor response (pre-clinical) in combination with checkpoint blockade. RGX-104 is in clinical trial for solid tumors as a single agent and in combination with PD-1 inhibitor (NCT02922764).	(90)

*CAR, chimeric antigen receptor; CTLA-4, cytotoxic T lymphocyte-associated antigen 4; DC, dendritic cells; iNKT, invariant NK T cell; irAEs, immune related adverse events; MDSC, myeloid-derived suppressor cells; NK, natural killer cells; PD-1, programmed death-1; PD-L1, programmed death ligand 1; TILs, tumor infiltrating lymphocytes*.

### Restricted T Cell Diversity and Exhausted Pro-inflammatory T Cell Response

Obesity can compromise T cell generation through thymic aging, thereby inhibiting T cell proliferation, compromising progenitor pools, and restricting the T cell repertoire (26). A key factor triggering this thymic involution is the transformation of thymic fibroblasts into adipocytes due to lipid accumulation (27). The decrease in thymic fibroblasts results in a decreased production of Stem cell factor (SCF), Fibroblast Growth Factor 7 and 10 (FGF7, FGF10), and Vascular Endothelial Growth Factor (VEGF) that are involved in thymocyte proliferation, thymic epithelial cell growth, and thymus vascularization. The concomitant increase in adipocytes leads to an increase in leukemia inhibitory factor (LIF), oncostatin M (OSM), and IL-6 that inhibit thymic function and trigger thymocyte apoptosis, thus compromising the T cell progenitor pool (28). Furthermore, T cells in obese individuals display an “exhausted” phenotype as a result of chronic inflammation (29) due to prolonged stimulation of toll-like receptors by circulating free fatty acids, activated stress responses, hypoxia, and adipocyte cell death (30). More specifically, functional skewing of T cells in pro-inflammatory CD8+, Th1 CD4+, and Th17 CD4+ cells, albeit exhausted, and downregulation of anti-inflammatory Th2 CD4+ and T regulatory (Treg) cells characterize the T cell response in obese subjects. Moreover, Tregs associated with obesity and hyperinsulinemia are deregulated with reduced IL-10 production and increased interferon gamma (IFN-γ) production, exacerbating meta-inflammation (31).

The obesity-associated immune dysregulation and dysfunction raises the question whether immune checkpoint blockade would be less effective in obese cancer patients or, in light of the increased T cell exhaustion, could more readily re-invigorate an anti-tumor immune response. Indeed, obese melanoma patients treated with targeted treatment or immune checkpoint inhibitors experienced improved overall and progression-free survival as compared to lean patients (32). A cross-species study provided some mechanistic insight as obesity was associated with PD-1 upregulation, impaired proliferation and an “exhausted” T cell molecular signature (29). Obesity correlated with better response to PD-1/PD-L1 blockade in tumor-bearing mice, as demonstrated by an increase in TILs and CD8+/CD4+ ratio, reduction in tumor burden and metastases, and improved survival. Moreover, analysis of 250 cancer patients treated with PD-1/PD-L1 blockade revealed a significantly better progression-free and overall survival in obese vs. lean patients. Of note, no increase in irAEs was observed, suggesting that obesity could be safely used as a biomarker to stratify patients for treatment with checkpoint inhibitors. In contrast, treatment response to CTLA-4 blockade was not improved in diet-induced obese mice (33).

How the paradoxical relationship between obesity-associated immune dysfunction and improved treatment response plays out in TNBC remains to be determined. It is likely that obesity, akin to PD-L1 expression, is a marker of immune suppression but also a marker of opportunity for immune intervention. The JAVELIN clinical trial reported a higher overall response rate to PD-L1 inhibition in metastatic TNBC patients with PD-L1+ TILs (34). Although the expression of PD-1/PD-L1 is heterogeneous and relatively low in TNBC, the JAVELIN findings suggest that obese TNBC patients with an exhausted immune response might benefit from checkpoint blockade (35). In addition, the increased infiltration of Tregs, CD8+ T cells, and CD20+ B cells in the TNBC tumor microenvironment implies that reversal of T cell exhaustion by checkpoint inhibitors could induce a strong anti-tumor immune response in obese patients (36). Furthermore, it has been reported that pro-inflammatory cytokines stimulate the expression of neo-epitopes, which can subsequently elicit a strong T cell response (37).

### Polarization of Gamma Delta (γδ) T Cells

Gamma delta (γδ) T cells form a subgroup of T cells that comprise 1–5% of circulating T cells. They are defined by heterodimeric T cell receptors (TCR) composed by γ and δ chains as opposed to α and β chains that make up the classical TCR of CD4+ and CD8+ T cells. The ability of γδ T cells to recognize abnormal cells in a MHC-unrestricted context together with the production of cytokines and chemokines (IFN-γ, IL-17, RANTES) highlighted their potential use for cellular immunotherapy. However, recent evidence has dampened the enthusiasm to use γδ T cells for adoptive cell therapy as several subsets have been identified that display pro-tumorigenic behavior. Although, Vγ9Vδ2 T cells with anti-tumor activity represent the predominant subset of activated γδ T cells in peripheral blood, microenvironmental cues can polarize resting γδ T cells into immunosuppressive, pro-tumorigenic FoxP3+ γδ Treg cells or γδ T17 cells (38). More specifically, increased IL-23, IL-6, IL-1β, and transforming growth factor-beta (TGF-β) levels induce polarization into γδ Tregs, impairing T cell and dendritic cell function; while IL-15 and TGF-β stimulation induce γδ T17 cells, promoting myeloid derived suppressor cells (MDSC) (38). This plasticity could in part explain why γδ T cells have been associated with poor prognosis in breast cancer (39) and why the results from γδ T cell-based immunotherapy have been rather disappointing (38). While the safety of γδ adoptive immunotherapy has been established, the efficacy remains low with response rates of 21% across cancers and 30% in breast cancer (38). A clinical trial using Zoledronate, a Vγ9Vδ2 T cell agonist, along with low-dose IL-2 demonstrated improved clinical outcome in metastatic breast cancer patients with a sustained population of mature Vγ9Vδ2 T cells (40). Patients with a decline in the number of Vγ9Vδ2 T cells experienced a worse outcome, which could be related to γδ T cell anergy or to an increased polarization into immunosuppressive γδ T cells. The latter being supported by a study on squamous cell carcinoma that reported a shift toward γδ T17 cells in more advanced disease (41). Furthermore, the success of γδ T cell therapy might also be impeded by an increased expression of PD-L1, thereby inhibiting activation of cytotoxic αβ CD8+ T cells as shown in pancreatic ductal adenocarcinoma (42).

In line with an exhausted immune response, obesity has been shown to trigger pro-inflammatory Vγ4 and Vγ6 γδ T cell accumulation in mouse adipose tissue (43), while reducing the number and functionality of circulating anti-tumorigenic Vγ9Vδ2 T cells (44, 45). The decreased activity of Vγ9Vδ2 T cells has been suggested to result from decreased IFN-γ production due to reduced circulating IL-2 levels and IL-2 receptor expression in obese individuals (44). However, maintaining γδ T cell activation by IL-2 supplementation would only be beneficial if we can overcome the obstacle to obtain persistent anti-tumor Vγ9Vδ2 T cell populations. As obesity increases the levels of IL-23, IL-6, IL-1β, and TGF-β (17), we can envisage that the efficacy of γδ T cell-based immunotherapy may not only be reduced in obese breast cancer cases but may even prove detrimental by polarizing the γδ T cells into their pro-tumorigenic γδ Treg and γδ T17 counterparts. Further studies are needed to investigate the biology of γδ T cells in breast cancer, specifically TNBC, and its correlation with obesity.

### Reduced Anti-tumor Function of Natural Killer Cells

Natural killer (NK) cells are integral to innate immunity and can be subclassified into CD56^dim^ cells that are more specialized in cytotoxicity through the production of granzyme and perforin, and CD56^bright^ cells that are bestowed with a regulatory function through the production of immunomodulatory cytokines (46). Breast cancer progression is associated with impaired NK cytotoxicity via downregulation of activating receptors (NKG2D, DNAM-1, CD16, CD69) and upregulation of inhibitory receptors (NKG2A) (47). In addition, a low number of NK cells together with a high number of tumor associated macrophages (TAMs) has been correlated with poor survival in TNBC (48), possibly due to the inhibitory effect of TAM-derived growth arrest-specific protein 6 (Gas6) on the anti-metastatic NK cell activity (49). Poor prognosis in TNBC has also been linked to an increase in immature, non-cytotoxic NK cells (CD56^dim^CD16^dim/−^) with increased PD-1 expression; and a decrease in mature, cytotoxic NK cells (CD56^dim^ CD16^bright^) (50).

In obesity, NK (CD3-CD56+) and NKT cells (CD3+CD56+) that have features of NK and T cells and play a role in tumor immunosurveillance (51), are significantly downregulated in number as well as in activity (52). Furthermore, obese individuals display a shift from more cytotoxic CD56^dim^ cells to less cytotoxic, immunomodulatory CD56^bright^ cells, resulting in chronic inflammation and increased cancer risk (53). In line with this, NK-markers of cytotoxicity such as TNF-related apoptosis-inducing ligand (TRAIL) and CD107a are reduced (54). In addition, chronic elevated levels of leptin desensitize the Ob-R leptin receptor on NK cells, reducing their activity through decreased phosphorylation of the JAK2 pathway and IFN-γ production (54).

It is likely that TNBC patients with obesity could experience a synergistic reduction in NK cell numbers and functionality. One approach to counteract this would be to use adoptive NK cell therapy. Preclinical work suggests that cell transfer of the invariant NK T cell (iNKT) population in obese subjects could increase IFN-γ secretion by NK cells. Adoptive transfer or activation of iNKT cells in obese mice reduced body weight, decreased triglyceride and leptin levels while increasing the production of anti-inflammatory cytokines that could counterbalance the meta-inflammatory state and enhance the anti-tumor immune response (55). A second strategy to boost the anti-tumor immune response in obese cancer patients could involve checkpoint inhibition of CD56^dim^CD16^dim/−^ NK cells with high PD-1 expression. Although the role of PD-1 in NK cell activity is currently not well-established, one preclinical study showed that PD-1/PD-L1 inhibition induced NK cell-mediated tumor regression (56). A third strategy could involve the selective ablation of a distinct subset of IL-6 receptor+ (IL6Ra+) NK cells and/or inhibition of IL-6 signaling in obese patients. These therapeutic interventions could possibly restore immunity as IL6Ra+ NK cell ablation in mouse models resulted in reduced obesity, meta-inflammation, and insulin resistance (57). Hence, combining NK cell therapy with checkpoint blockade may prove beneficial in obese cancer patients.

### Impaired Efficacy of Dendritic Cell-Dependent Anti-tumor Immunity

Dendritic cells (DCs) are the professional antigen-presenting cells that prime and stimulate naïve T cells into effector cells. DCs can be subclassified as myeloid or plasmacytoid based on their bone marrow precursors. The number of peripheral myeloid DCs is downregulated in breast cancer, leading to reduced levels of IL-12, impaired DC maturation, and hampered immunosurveillance (58). In contrast, plasmacytoid DCs, deficient in IFN-α, are enriched in TNBC tumors and contribute to Treg expansion, immunosuppression, and poor prognosis (59). The use of the DC-based vaccine Provenge^TM^ has been FDA-approved for the treatment of prostate cancer, while the GVAX vaccine is currently in clinical trial for the treatment of pancreatic cancer. In breast cancer, treatment with Her2 peptide-pulsed DCs resulted in pathological complete responses in pre-invasive Her2+ tumors (60).

To the best of our knowledge, the effects of obesity on the efficacy of DC-based immunotherapy has not been studied in humans. Using a diet-induced obesity mouse model, DC-based immunotherapy with Adenovirus-encoded TRAIL (AdTRAIL) reduced the tumor growth of renal cell carcinoma in lean mice, but failed to decrease tumor outgrowth in obese mice (61). The lack of tumor response in obese mice was accompanied by an increase in regulatory DCs and a decrease in tumor infiltration of IFN-γ-producing CD8+ T cells. Another study reported that the majority of myeloid DCs in obese subcutaneous adipose tissue are pro-inflammatory CD11c+CD1c+ cells that induce Th17 effector cells and IL-17 production, and correlate positively with BMI and insulin resistance (62). The dampened response to DC-vaccines in obese mice could additionally be related to obesity-mediated upregulation of TGF-β that can affect DC functionality, including maturation, IL-12 secretion, migration, antigen presentation, and stimulation of T cells (63). Furthermore, autologous DC-based immunotherapy, whereby patient-derived DCs undergo *ex vivo* manipulation to express tumor-associated antigens is likely to be inefficient due to the suppressive and exhausted nature of obesity-associated DCs.

### Altered Macrophage Polarization

The binary spectrum of macrophages comprises the classical M1 and alternative M2 type macrophages. While Lipopolysaccharide (LPS) and IFN-γ primarily mediate activation of macrophages into pro-inflammatory M1 macrophages; IL-4 and IL-13 polarize macrophages into the anti-inflammatory M2 phenotype (64). During tumor development and progression, circulating bone marrow-derived monocytes are recruited to the tumor by chemokine C-C motif ligand 2 (CCL2) and macrophage colony stimulating factor (M-CSF)-1 where they differentiate into TAMs with a M2-like phenotype (65). These M2-like cells exhibit pro-tumorigenic features, supporting angiogenesis, epithelial-to-mesenchymal transition, cell migration and invasion, and intra- and extravasation through the secretion of pro-angiogenic and pro-migratory factors, and proteolytic enzymes (66). In accordance, an increase in TAMs has been associated with poor clinical outcome of high grade, hormone receptor negative, basal-like subtypes of breast cancer (67). Thus, the development of macrophage-specific anti-tumor strategies has involved interference with polarization and elimination of M2 macrophages. For instance, epigenetic modulators have been used to suppress M2 polarization or to induce M1 polarizing- gene expression, while accumulation of M2 macrophages has been impeded through inhibition of CCL2/CCR2 and CSF-1/CSF-1R or by triggering TRAIL-mediated apoptosis (68). Further studies are necessary to identify biomarkers to target specifically TAMs without affecting the M1 macrophage pool. In this regard, a murine TAM-specific peptide (M2pep) has been identified that preferentially targets M2 polarized TAMs with high affinity and can be fused with pro-apoptotic peptides (69). Furthermore, targeting the MAcrophage Receptor with COllagenous domain (MARCO) receptor on TAMs in solid tumor mouse models specifically reduced the frequency of M2 macrophages that express arginase-1 (ARG-1) and suppress T cell proliferation (70). On another note, similar to M2 macrophages, M2-like TAMs inhibit CD8+ signaling and function through the secretion of immunosuppressive molecules (IL-10, TGF-β, ARG-1, prostaglandins) (66). In addition, TAMs express the targetable immune checkpoint ligands PD-L1/PD-L2 and CD80/CD86 (71). In this respect, preclinical studies have demonstrated additional benefit from combining checkpoint blockade with strategies to intervene with TAM accumulation, polarization, and/or function (72).

It is important to consider the functions of adipose tissue macrophages in addition to circulating macrophages in relation to invigorating inflammation in adipose and peripheral tissues. Indeed, obesity is characterized by an increased macrophage recruitment in adipose tissue (45–60% in obese vs. 10–15% in lean subjects) resulting in crown-like structures (CLS) arranged around dead adipocytes due to excessive fat accumulation (73). CLS formation is associated with the production of TNF-α, inducible nitric oxide synthase (iNOS), CCL2 and IFN-γ; which are stimuli for M2 to M1 polarization via NF-kB and signal transducer and activation of transcription (STAT)-1 signaling (74). Elevated LPS and reduced adiponectin levels further support the recruitment and activation of M1 macrophages (75). Moreover, the overall immune landscape in the obese adipose tissue niche drastically changes in favor of M1 macrophage enrichment. More specifically, CD8+ T cells, IFN-γ producing Th1 cells, neutrophils, B cells, and NK cells can stimulate M1 macrophage polarization, infiltration, and activation (76). However, recent transcriptomic and proteomic analyses have suggested that using a binary M1/M2 classification in obesity is an oversimplification (77).

Whether the pro-inflammatory adipocyte niche in obesity (containing M1 macrophages) prevails over the immunosuppressive M2 infiltration in cancer, and how the pro-tumorigenic features of these M2 macrophages can be counterbalanced in tumors remains to be determined.

### Accumulation of Myeloid-Derived Suppressor Cells

Myeloid-derived suppressor cells form a heterogeneous population of bone marrow-derived myeloid cells that fail to differentiate into mature myeloid lineages such as macrophages and DCs. They are subclassified as monocytic MDSCs (M-MDSC) with a CD11b^+^CD14^+^CD15^−^HLA-DR^low/−^ phenotype and polymorphonuclear/granulocytic MDSCs (PMN-MDSC) phenotyped as CD11b^+^CD14^−^CD15^+^HLA-DR^−^ (78). MDSCs are important regulators of immune suppression via inhibition of T cells, NK cell cytotoxicity and macrophage polarization. Their T cell immunosuppressive potential is mediated by ARG1, iNOS, and indoleamine 2,3-dioxygenase (IDO), which inhibit T cell proliferation and TCR signaling while inducing T cell anergy and promoting Treg differentiation (78). In addition, increased expression of PD-L1 and cell death receptor Fas/CD95 mediate T cell suppression through binding to PD-1 and Fas-L on T cells (79, 80). Several studies have demonstrated MDSC accumulation in the circulation and the tumor microenvironment of breast cancer patients, in particular in metastatic breast cancer (81, 82).

A recent murine study reported a novel mechanism linking obesity, immune suppression, and tumor progression. They found that obesity induced tumor infiltration of MDSCs as well as MDSC expression of PD-L1 thereby inhibiting anti-tumor responses and promoting tumor growth, metastasis, and poor prognosis (83). Mobilization of M-MDSCs and PMN-MDSCs from the bone marrow into the circulation and tumor site is mediated by the CCR2-CCL2 and CCR5-CCL3/4/5 axis, respectively (84). Hence, obesity-associated increases in CCL2 and CCL5 levels, together with an increase in estrogen and pro-inflammatory mediators such as leptin, IL-6, IL-1β, and TNF-α could facilitate MDSC accumulation (85–87). CCL5 was also found to support the differentiation and immunosuppressive activity of MDSCs in the 4T1 TNBC mouse model, and hence to promote tumor progression (88). Interestingly, increased glycolytic activity of murine TNBC tumors was associated with a high MDSC density and poor survival, which could be reversed by glycolysis restriction (89). A recent study provided evidence of tumor regression after treatment with MDSC-targeting agents. The authors found that activation of Liver X receptor/Apolipoprotein E (LXR/ApoE) signaling by LXRβ agonists (GW3965 and RGX-104a) induced MDSC apoptosis, alleviating their immunosuppressive effect on cytotoxic T cell activity (90). In addition, LXR targeting in combination with checkpoint blockade significantly augmented the anti-tumor immune response in preclinical models. The more potent agonist RGX-104 is now in clinical trial for treatment of solid tumors (including TNBC) as a single agent or in combination with the PD-1 inhibitor Nivolumab (NCT02922764). In addition, it would be of interest to investigate whether intervention for obesity-associated hormone imbalances in combination with MDSC-targeting drugs could enhance anti-tumor immunity. Indeed, it has been shown that the immunosuppressive function of MDSCs relies on the uptake and accumulation of exogenous lipids, which increases their ARG1 and iNOS expression and inhibits T cell cytotoxicity via a STAT3/STAT5-dependent pathway (91).

## Future Prospects in Obesity-Associated Cancer Immunotherapy

Generally, breast tumors are characterized by a relatively low T cell infiltration and lack of response to checkpoint inhibitors. Although TNBC might be the least “immunologically cold” breast cancer subtype, many factors can still contribute to dampening anti-tumor immunity and immunotherapy efficacy. For example, poor T cell priming, “exhaustion” and impaired expansion can result from low-grade chronic inflammation as observed in obesity. Although the deregulatory effects of obesity on the immune system and cancer progression have been established, the majority of immunotherapy clinical studies overlook the baseline immune disposition of a patient. Based on our current knowledge, we can foresee several challenges and opportunities for the clinical management of cancer immunotherapy in obese patients.

Cancer immunotherapy focuses on augmenting the anti-tumor immune response, which could reinvigorate the meta-inflammatory state of obese patients to an extent that results in cytokine storms and irAEs. Similar hyperactivation of the immune system has been demonstrated in aging mice with increased visceral adiposity where systemic immunotherapy with anti-CD40 and IL-2 triggered a cytokine storm, increased adipose M1 macrophage polarization and induced release of pro-inflammatory TNF-α and IL-6 resulting in reduced anti-tumor efficacy and worse survival. Calorie-restriction, macrophage depletion and TNF-α blockade could reverse the excess toxicity in this mouse model (92, 93). Likewise, whereas CTLA-4 checkpoint blockade is used to increase anti-tumor immunity; this approach could be contraindicative in obese patients due to excess activation of the chronic low-grade inflammation. Instead, the CTLA4-Ig fusion protein abatacept has been used as an agonist to bind CD80 and CD86, thereby reducing the availability of CD80/CD86 to bind to CTLA-4 (preventing the T cell inhibitory pathway similar to anti-CTLA-4 antibodies) as well as CD28, resulting in the inhibition of T cell activation (94). In diet-induced obese mice, treatment with CTLA4-Ig reduced body weight, improved insulin resistance and reduced adipose tissue inflammation by a mechanism involving adiponectin upregulation, M1 to M2 macrophage polarization, Treg stimulation, and TGF-β signaling (95, 96). Thus, a key challenge in immunotherapy is to augment anti-tumor immunity without exacerbating the pro-tumorigenic obesity-associated inflammation, which may require different approaches for the same target as discussed above for CTLA-4.

Obesity-associated immune perturbations, however, can also be found in cancer patients and can therefore be targeted simultaneously in obese cancer patients. For example, TGF-β levels are increased in advanced tumors as well as in obesity and hence, obese cancer patients may not be able to sustain an effective T cell response after adoptive T cell therapy. Although the development of new generation Chimeric Antigen Receptor (CAR)-T cells with co-stimulatory domains has improved T cell persistence, survival, and cytokine secretion; tumors can inhibit the anti-tumor potency of these cells through the TGF-β pathway. Upregulation of TGF-β has been shown to impair CAR-T cell proliferation and activity, and to downregulate intracellular levels of perforin, GM-CSF, and IFN-γ (97). Since several initiatives are undertaken to test CAR-T cell therapy in TNBC (98), it is important to explore the effect of blocking TGF-β1 signaling on treatment response. Moreover, recent *in vivo* studies have indicated that combining TGF-β1 blockade with different immunotherapy modalities can significantly improve the anti-tumor immune response. For example, anti-PD-L1 therapy in combination with a TGF-β1 blocking antibody significantly increased tumor infiltration of CD8+ T cells and increased tumor regression (99). A similar observation was made using a bifunctional fusion protein (M7824) comprising of the extracellular domain of the TGF-β receptor and the C-terminus of the anti-PD-L1 heavy chain, thus blocking both TGF-β1 and PD-1 signaling (100). Thus, it seems critical to include obese cancer patients in TGF-β blockade clinical trials in order to dissect the impact of obesity-associated TGF-β1 signaling on immunotherapy response.

Not only can we target shared obesity- and cancer-related immune dysregulations, we can also exploit the obesity-associated immune dysfunction to heighten cancer immunotherapy efficacy. A recent retrospective study of metastatic melanoma patients demonstrated improved progression-free and overall survival in obese male patients treated with checkpoint inhibitors as compared to patients with normal BMI (32). This paradoxical observation was corroborated in a cross-species cancer study, although irrespective of gender (29). Hence, obesity promotes tumor progression and immune dysfunction, in particular by PD-1 upregulation; and this phenomenon can be exploited to improve response to PD-1/PD-L1 inhibition. This paradoxical effect of obesity on tumorigenesis and response to immune checkpoint inhibitors needs to be validated in TNBC using cohorts with sufficient statistical power to overcome the effects of additional confounding factors.

## Concluding Remarks

In conclusion, we believe it is imperative to include obese cancer patients in TNBC immunotherapy clinical trials in order to accurately determine treatment safety and efficacy. In addition, murine models should further explore the molecular mechanisms by which obesity can affect anti-tumor immunity and efficacy of immune checkpoint inhibitors. This knowledge will pave the path for and necessitate personalized immunotherapy. Incorporating immunotherapy, chemotherapy and lifestyle intervention in the standard of care for obesity-associated TNBC may provide optimistic prospects to improve prognosis and survival.

## Author Contributions

JD conceived and critically revised the manuscript. AN drafted the manuscript and designed the figures and tables. AM critically revised the manuscript and contributed to writing the final manuscript. All authors read and approved the manuscript for publication.

### Conflict of Interest Statement

AM serves on the advisory board of AstraZeneca, Dynavax and Incyte and has received research funding from Genetech, Merck, Incyte, EMD Serono, Transgene, and BMS. The remaining authors declare that the research was conducted in the absence of any commercial or financial relationships that could be construed as a potential conflict of interest.
